# Continuous respiratory rate monitoring during an acute hypoxic challenge using a depth sensing camera

**DOI:** 10.1007/s10877-019-00417-6

**Published:** 2019-11-08

**Authors:** Paul S. Addison, Philip Smit, Dominique Jacquel, Ulf R. Borg

**Affiliations:** 1 Medtronic, Video Biosignals Group, Patient Monitoring, Technopole Centre, Edinburgh, EH26 0PJ UK; 2 Medtronic, Medical Affairs, Patient Monitoring, Boulder, CO USA

**Keywords:** Non-contact monitoring, Depth sensing, Respiratory rate, Hypoxic challenge

## Abstract

Respiratory rate is a well-known to be a clinically important parameter with numerous clinical uses including the assessment of disease state and the prediction of deterioration. It is frequently monitored using simple spot checks where reporting is intermittent and often prone to error. We report here on an algorithm to determine respiratory rate continuously and robustly using a non-contact method based on depth sensing camera technology. The respiratory rate of 14 healthy volunteers was studied during an acute hypoxic challenge where blood oxygen saturation was reduced in steps to a target 70% oxygen saturation and which elicited a wide range of respiratory rates. Depth sensing data streams were acquired and processed to generate a respiratory rate (RR_depth_). This was compared to a reference respiratory rate determined from a capnograph (RR_cap_). The bias and root mean squared difference (RMSD) accuracy between RR_depth_ and the reference RR_cap_ was found to be 0.04 bpm and 0.66 bpm respectively. The least squares fit regression equation was determined to be: RR_depth_ = 0.99 × RR_cap_ + 0.13 and the resulting Pearson correlation coefficient, R, was 0.99 (p < 0.001). These results were achieved with a 100% reporting uptime. In conclusion, excellent agreement was found between RR_depth_ and RR_cap_. Further work should include a larger cohort combined with a protocol to further test algorithmic performance in the face of motion and interference typical of that experienced in the clinical setting.

## Introduction

The clinical importance of respiratory rate (RR) is well known as it provides important information regarding many aspects of a patient’s respiratory status. Changes in RR are often one of the earliest and more important indicators that precedes major clinical manifestations of serious complications such as respiratory tract infections, respiratory depression associated with opioid consumption, anaesthesia and/or sedation, as well as respiratory failure [1–3]. A wide range of methods have been proposed for the determination of respiratory rate using non-contact means including RGB video camera systems [4, 5], infrared camera systems [6], laser vibrometry [7], piezoelectric bed sensors [8], doppler radar [9], thermal imaging [10] and acoustic sensors [11]. The determination of respiratory information from depth data has received relatively less attention than RGB video methods, although such systems are well suited to this task.

The inflation and deflation of the lung during a respiratory cycle is a phenomenon measurable with a depth camera. Many studies have compared tidal volume, as measured by a reference system (e.g. a spirometer), with the tidal volume extracted by a depth camera system based on morphological changes in the chest wall. One of the earliest tidal volume measurements extracted in this way was carried out by Yu et al. [12]. They assessed a Kinect V1 based system against a spirometer and achieved a correlation coefficient, R = 0.97 (p < 0.001), based on 12 healthy subjects undertaking a range or respiratory activities including shallow, middle and deep breathing as well as isovolume maneuvers. Aoki et al. [13] performed a similar study using a Kinect V1 system comparing the tidal volumes to that measured using a gas analyser. Four healthy subjects were monitored over a 180 s acquisition period and they obtained an R = 0.99 correlation coefficient. A more recent study by Seppänen et al. [14] evaluated depth camera extracted tidal volume over a range of respiratory rates. Part of the data was used to train a bank of FIR filters from which the depth data volume was extracted. Various filter configurations were tested. Their best model produced R^2^ = 0.932 (R = 0.965) and a tidal volume accuracy error of 9.4%. In another study, Soleimani et al. [15] obtained volume and flow signals by processing Kinect V2 depth data to produce parameters typically associated with a pulmonary function testing. They evaluated their system on 40 COPD patients. Using a spirometer as a reference, they demonstrated that the forced vital capacity and slow vital capacity test both produced correlation coefficients of 0.999, (although they included a rescaling based upon the spirometry measurements to achieve this). Harte et al. [16] used four Kinect V1 cameras arranged in a cross configuration pointing towards the subject located in the centre. Their study included thirteen healthy subjects and nine cystic fibrosis patients. Based upon a one-way ANOVA test (F[1,51] = 7.5783; p = 0.0082), they concluded there was a significant difference in the sample means of the two groups. Transue et al. [17] evaluated a Kinect V2 based system against a spirometer for tidal volume accuracy. Their system performed a general volume estimation of the chest and then required per-subject training against a spirometer as reference to offer more accurate individual results. The dataset consisted of multiple trials of 20 s duration with participants in a standing position. They obtained accuracies ranging from 92.2 to 94.2% (corresponding to absolute errors in tidal volume from 0.055 to 0.079 l) when assessing four healthy subjects against a spirometer.

A number of research groups have focused specifically on determining a respiratory rate using depth sensing camera equipment. Bernacchia et al. [18] assessed ten healthy young adult subjects and found good agreement between the breath periods derived from a Kinect depth sensing system and a spirometer reference. They achieved a 9.7% RMSD for the breath periods between the two devices. During the tests, which lasted only 40 s per acquisition, the subjects were asked to maintain ‘regular respiratory activity’. In a study of young children (between 1 and 5 years) Al-Naji et al. [19] found excellent agreement between depth-sensing RR and a piezo-belt reference with correlation coefficients ranging from 0.97 to 0.99 depending on whether bed sheets were used and the background lighting levels. The study was, however, limited to five healthy volunteers in relatively benign conditions. Rezaei et al. [20] studied the respiratory rate of restrained rodents when subjected to fear-inducing predatory odours. They found they could measure respiratory rate with an accuracy of 94.8% using a reference RR from visual observation. Martinez and Stiefelhagen [21] utilised a depth camera to extract respiratory rate data from 94 sleep analysis sessions from 67 patients in a sleep clinic. They found their system to be 85.9% accurate when compared to a reference thermistor placed at the nose. However, their depth system achieved similar results to that of a contact chest band sensor. Monitoring the respiratory rates of three preterm infants was the focus of a study by Cenci et al. [22] where each infant was assessed in five 30 s intervals. They found an overall correlation coefficient of R = 0.95 between their system output and the respiratory rate derived from ECG impedance pneumography.

Much of the early work involving depth sensing cameras focused on the determination of tidal volume. More recent studies which have considered respiratory rate are, in general, limited to relatively benign conditions, short periods of time, limited numbers of subjects/patients and/or poor reference measures. The work reported here extends current research in this area by studying a cohort of healthy volunteers exhibiting a wide range of respiratory rates resulting from being subjected to a rigorous, protocolized hypoxic challenge.

## Methods

### Clinical study

The data was collected opportunistically during a non-related hypoxia (‘breathe-down’) study to evaluate a pulse-oximeter sensor. This parallel study protocol includes a desaturation event comprising a series of step-changes in oxygen saturation. Approval was given for the use of depth camera data acquisition and no other alteration to the existing protocol was made.

**Fig. 1:**
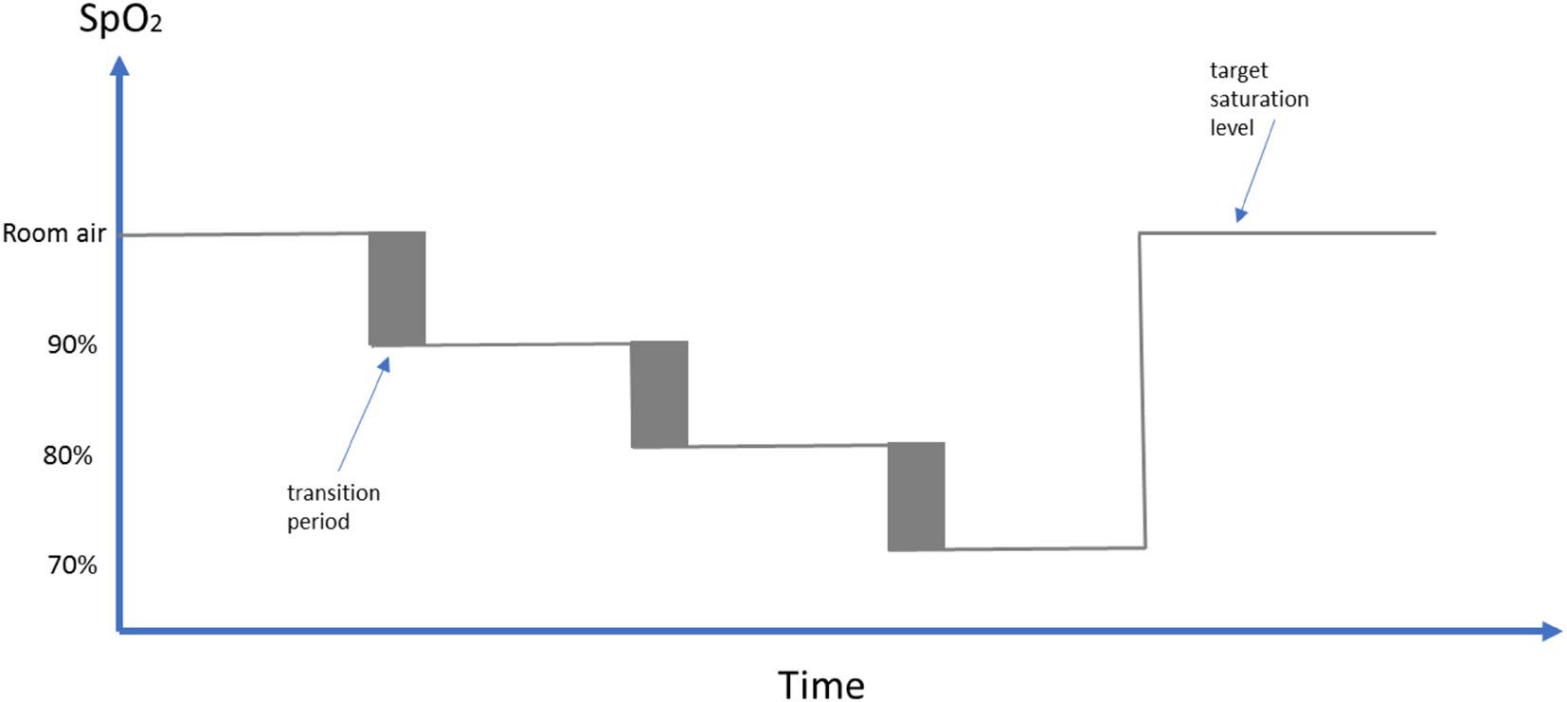
Desaturation

Fourteen subjects participated in the study. Subjects provided an institutional review board (IRB) approved informed consent covering the essential information stated in the protocol, as required elements according to 21 CFR 812.150 for a non-significant risk medical device investigation. The subjects were fitted with a face mask in order to adjust the FiO_2_ using a mixture of nitrogen and oxygen and induce desaturation. Each subject underwent a discrete episode of hypoxia. The sequence of targeted oxygen saturation levels is shown schematically in Fig. 1. In addition to the pulse oximetry data, capnography data was recorded during the study using a Datex-Ohmeda S/5 Monitor (GE Healthcare, Chicago, IL, USA). The capnograph is the reference against which we assessed our non-contact respiratory rate algorithm. Each session took approximately 35 min.

All 14 subjects successfully completed the hypoxic challenge. Due to technical reasons the primary study only captured 12 of the 14 desaturation profiles. Our secondary study however successfully captured depth information for all subjects, and since our study did not require the saturation data, we could analyse all 14 subjects. The subjects had a mean age of 31.9 (standard deviation, SD 6.9) years and mean body mass index (BMI) of 26.3 (SD 4.2). Individual demographic information is provided in Table 1. Exclusion criteria included subjects with known respiratory conditions and/or heart or cardiovascular conditions.

**Table 1:** Participant demographic information

Subject ID	Gender	Weight (kg)	Height (m)	BMI (%)	Age
001	Female	57	1.55	23.4	29
002	Male	84	1.80	25.8	33
003	Male	91	1.78	28.7	31
004	Male	100	1.80	30.8	48
005	Female	92	1.63	34.7	26
006	Male	95	1.88	27	36
007	Female	88	1.78	28	27
008	Male	68	1.75	22.1	26
009	Female	68	1.55	28.3	31
010	Male	71	1.78	22.4	38
011	Female	62	1.52	26.6	29
012	Female	61	1.63	23.2	25
013	Male	91	1.78	28.7	26
014	Female	54	1.73	17.9	42
	Mean	77.3	1.71	26.3	31.9
	SD	15.8	0.11	4.2	6.9

### Data acquisition and processing

The depth data was captured using a Kinect V2 camera (Microsoft Corporation, Redmond, WA, USA) connected to a laptop and at a frame rate of 30 fps. The camera was mounted on a tripod and placed in front of each subject. The data was collected over several days and the distance between the camera and subjects varied between 1.2 and 2.0 m over the collection period and positioned vertically at approximately chest height. The subjects were seated in a slightly reclined position. The room was illuminated with standard ceiling mounted fluorescent lights. Other than starting and stopping the recording process no other intervention or calibration was required over the study period.

**Fig. 2:**
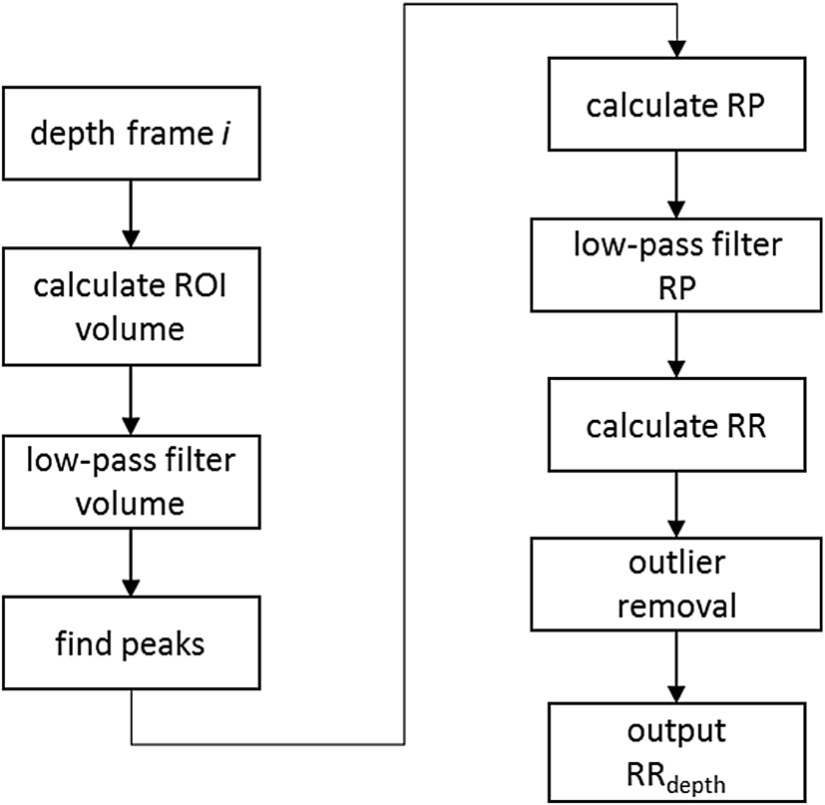
Algorithm flow diagram

**Fig. 3:**
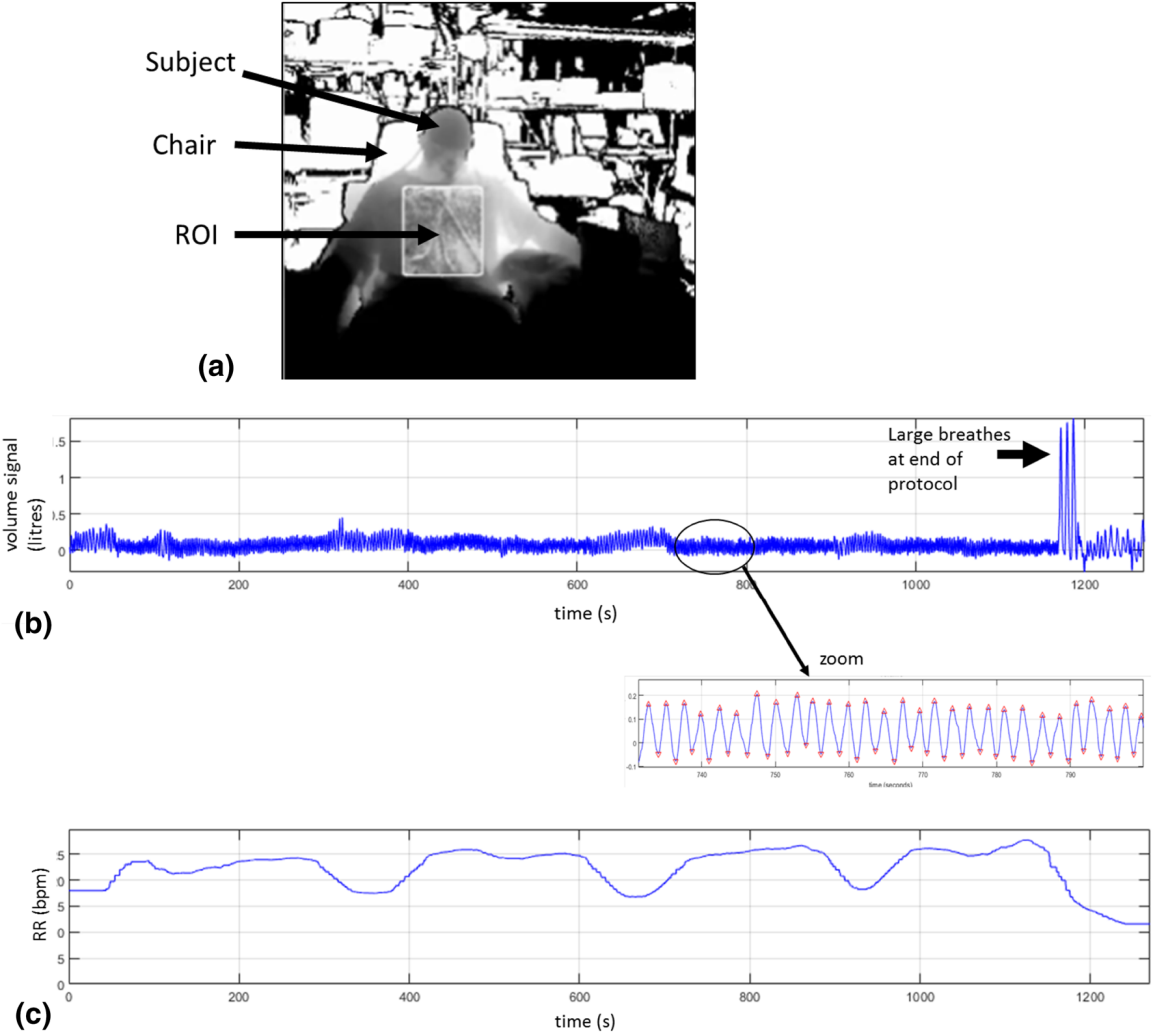
**a** Depth image with ROI. **b** Respiratory volume signal. Zoom shows respiratory modulations with peaks and troughs indicated. **c** RR_depth_

Respiratory rate (RR) is extracted algorithmically from the acquired depth data as illustrated in Fig. 2. A region-of-interest (ROI) is defined on the torso area of the subject (Fig. 3a). An estimate of the volume change across the ROI over time is obtained by calculating the depth changes of each frame and integrating spatially across the ROI. The resulting volume signal offers a clear indication of the breathing pattern as shown in Fig. 3b where the peaks and troughs of the individual breaths are marked. Note that Fig. 3b shows a whole trace from one of the subjects in the trial. Three large breaths are obvious in the main plot (marked by the arrow) at the end of the trial where the subject is instructed to take large breaths at the end of the hypoxic challenge. The zoomed-in portion of the signal shows the respiratory modulations in more detail.

The next steps in the algorithm (outlined in Fig. 2) extract a robust value of respiratory rate (RR) from the volume signal. The respiratory volume signal is first filtered by a low pass filter (Butterworth, 5th order, cut-off 0.67 Hz). The peaks of this signal are then identified and the respiratory periods (RPs) calculated as the time difference between successive peaks to produce a “per breath” RP signal. This RP signal is low-pass filtered (Butterworth, 5th order, cut-off 0.67 Hz) to smooth the periods. The RR signal is then calculated by multiplying the reciprocal of the RP signal by 60. The final step removes the effect of outliers in the RR signal by averaging over a 60 s sliding window only those points that are within the 25th and 75th percentiles of the values. (We have found that these outliers may arise if non-prominent peaks are not successfully eliminated during the initial stages of the algorithm and this outlier removal step successfully deals with these.) This processing produces the output RR_depth_ signal, an example of which is shown in Fig. 3c.

The capnograph provides a reference respiratory rate on a per-second basis. This output reporting time step duration is relatively typical for medical monitoring devices for screen updating. We therefore resampled the output of the depth sensing RR to match this. The two respiratory rate signals, RR_depth_ and RR_cap_, required synchronization prior to statistical analysis as the depth camera and capnograph signals were collected independently on separate acquisition systems. This was carried out using cross-correlation of the two signals.

### Data analysis

*Bias* and *accuracy* statistics were calculated to compare the depth data derived RR with that of the reference (capnograph) system. These are, respectively, the mean difference and the root mean squared difference (RMSD) between the test and reference values. That is

1 $$Bias = \frac{{\mathop \sum \nolimits_{i = 1}^{N} \left( {RR_{depth} \left( i \right) - RR_{cap} \left( i \right)} \right)}}{N}$$

and

2 $$RMSD\,accuracy = \sqrt {\frac{{\mathop \sum \nolimits_{i = 1}^{N} \left( {RR_{depth} \left( i \right) - RR_{cap} \left( i \right)} \right)^{2} }}{N}}$$

The latter expression is a root mean square deviation (RMSD) and represents a combination of the systematic and random components of the differences between the corresponding readings from the two devices.

Least-squares linear regression was performed to obtain the line of best fit between the video and reference parameters from which the gradient, intercept, Pearson correlation coefficient, R, and associated p values were computed. In this work p < 0.05 was considered statistically significant. A Bland–Altman analysis of the data was also performed using the method of Bland and Altman [23] which compensates for within-subject longitudinal correlation in the data. SD of the bias and corresponding limits of agreement were calculated using this methodology.

A reliability measure in the form of an *uptime* was computed. This is a measure of the percentage of time that RR_depth_ can be computed for each subject. A high uptime is usually a fundamental technical requirement for the development of a medical device. To be acceptable for use in clinical practice, both accuracy and uptime must be sufficiently high. Note that accuracy may be improved at the expense of uptime by avoiding posting results when the signal quality is poor (e.g., due to noise). We define uptime, here as the duration, T_valid_, that a valid respiratory rate can be calculated and reported by the algorithm as a percentage of the total acquisition time, T_acq_, i.e.

3 $$Uptime = \frac{{T_{valid} }}{{T_{acq} }} \times 100$$

Matlab (R2018b) was used to process the data and perform the statistical analysis. An in-house developed C++ application was used to capture the depth data.

## Results

**Fig. 4:**
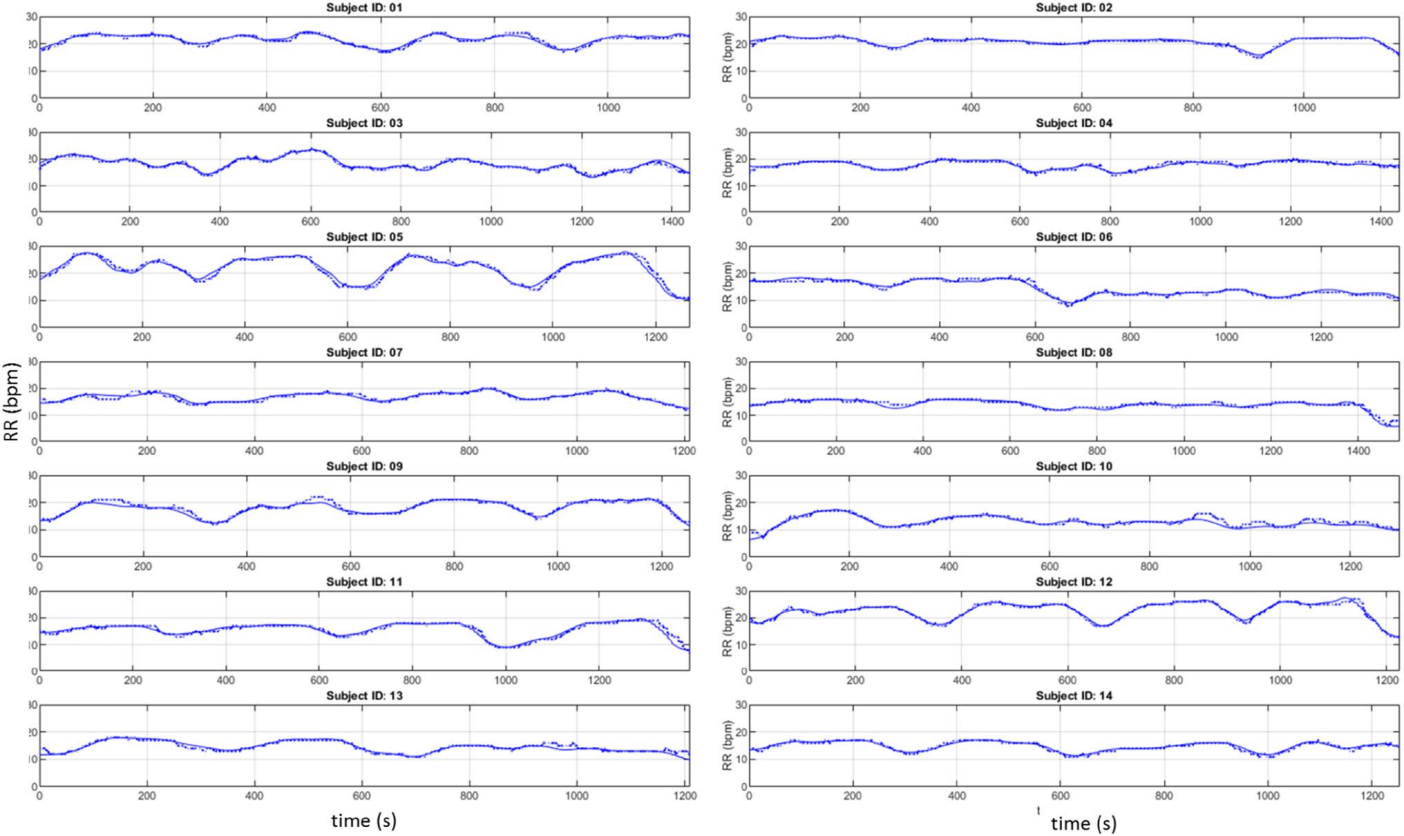
Time series of depth sensing and capnograph respiratory rates for each subject (RR_depth_ = solid, RR_cap_ = dotted)

Figure 4 contains the comparative RR signals for all fourteen subjects. The primary protocol did not state any specific breathing paradigm other than to breathe normally and to take deep breaths while the air supply was returned to room air at the end of the session.

**Fig. 5:**
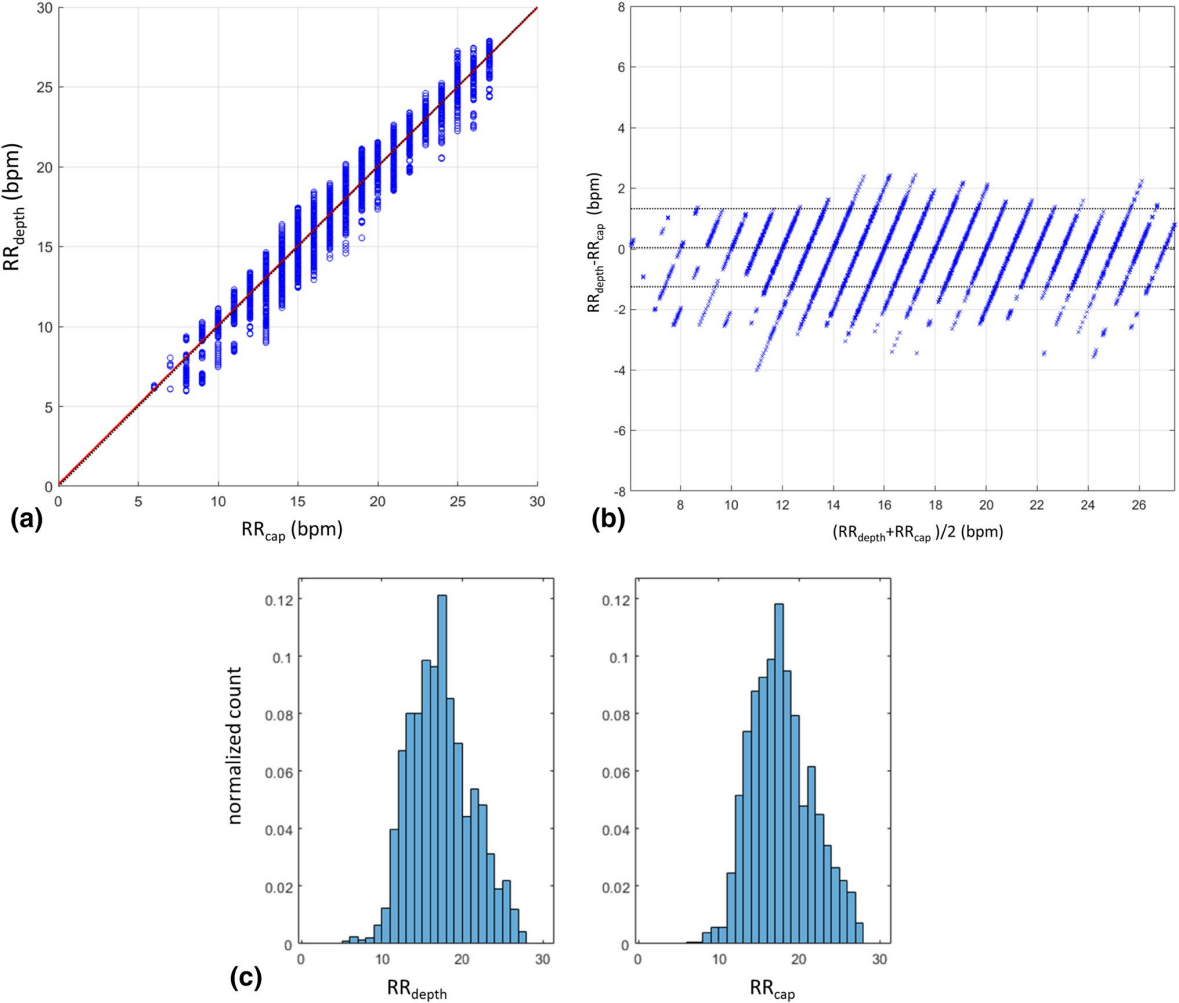
**a** Scatterplot of respiratory rates: depth sensing camera rates against capnography reference rates. **b** Bland–Altman plot of respiratory rates showing the mean bias and limits of agreement. **c** Respiratory rate distribution plots for the depth sensing camera and capnography reference

Figure 5a contains the scatterplot of RR_depth_ against RR_cap_ aggregated over all subjects. Here each point in the plot represents a respiratory rate calculated from an individual breath of one of the subjects. The bias and accuracy (RMSD) for all subjects pooled together was 0.04 bpm and 0.66 bmp respectively and with a Pearson’s correlation coefficient of 0.99 (p < 0.001) The corresponding Bland–Altman plot is shown in Fig. 5b for comparison and shows that 95% of calculated RR_depth_ values occur within 1.32 bpm of the reference system. The distributions of the individual respiratory rates across all subjects for the depth sensing and reference capnograph devices are shown in Fig. 5c. We can see that both distributions look very similar.

**Fig. 6:**
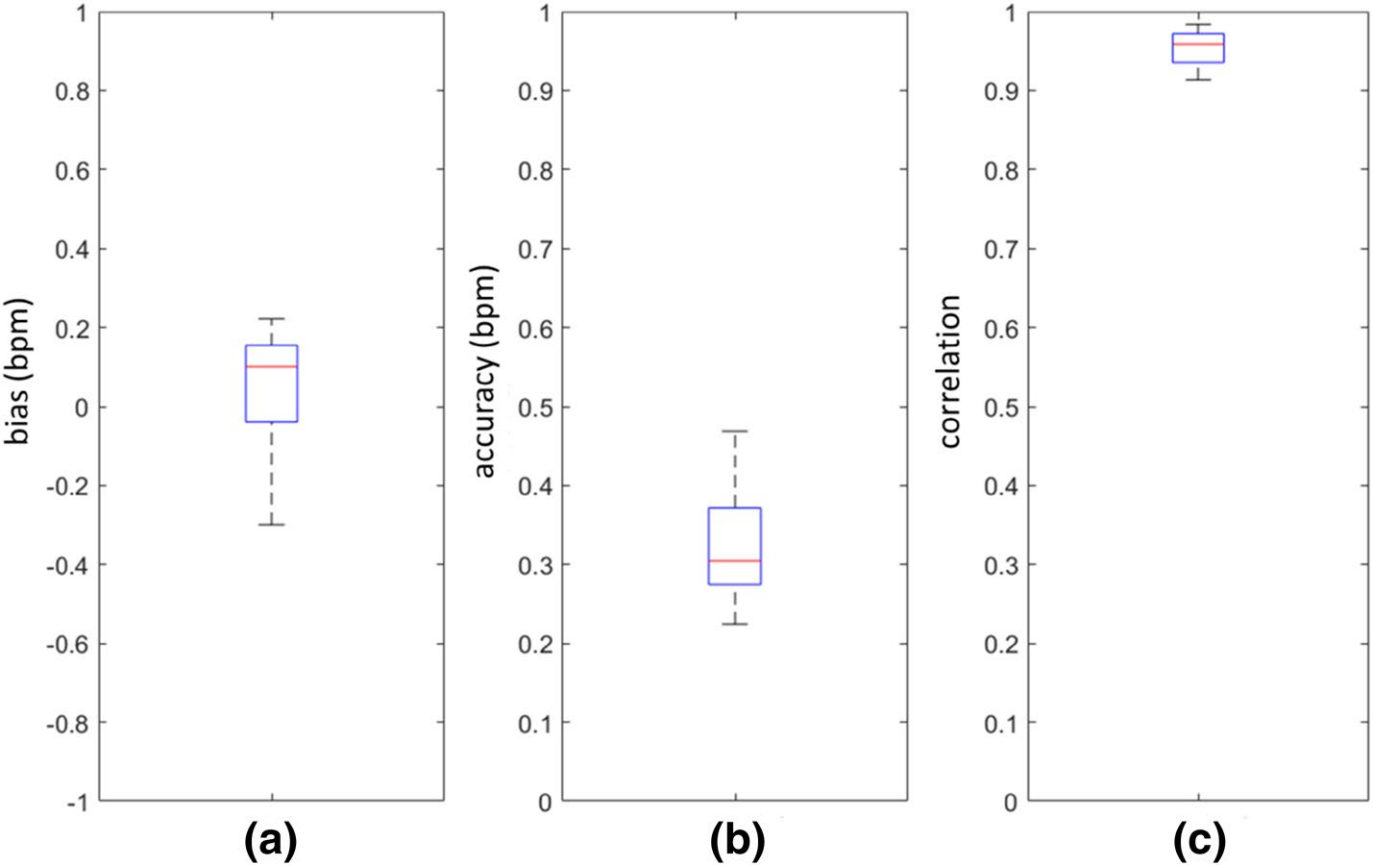
Boxplots of Per-Subject Statistics. **a** Mean bias. **b** RMSD accuracy. **c** Pearson correlation (R)

The data was also analysed on a per-subject basis. The box plots of Fig. 6a, b show the spread of the individual mean biases, which range from − 0.30 to 0.22 bpm, and the RMSD accuracies, which varies from 0.45 to 0.94 bmp. The spread of the individual Pearson correlation coefficients is shown in Fig. 6c which ranged from 0.91 to 0.98.

Uptime was calculated to be 100% for every case.

## Discussion

**Fig. 7:**
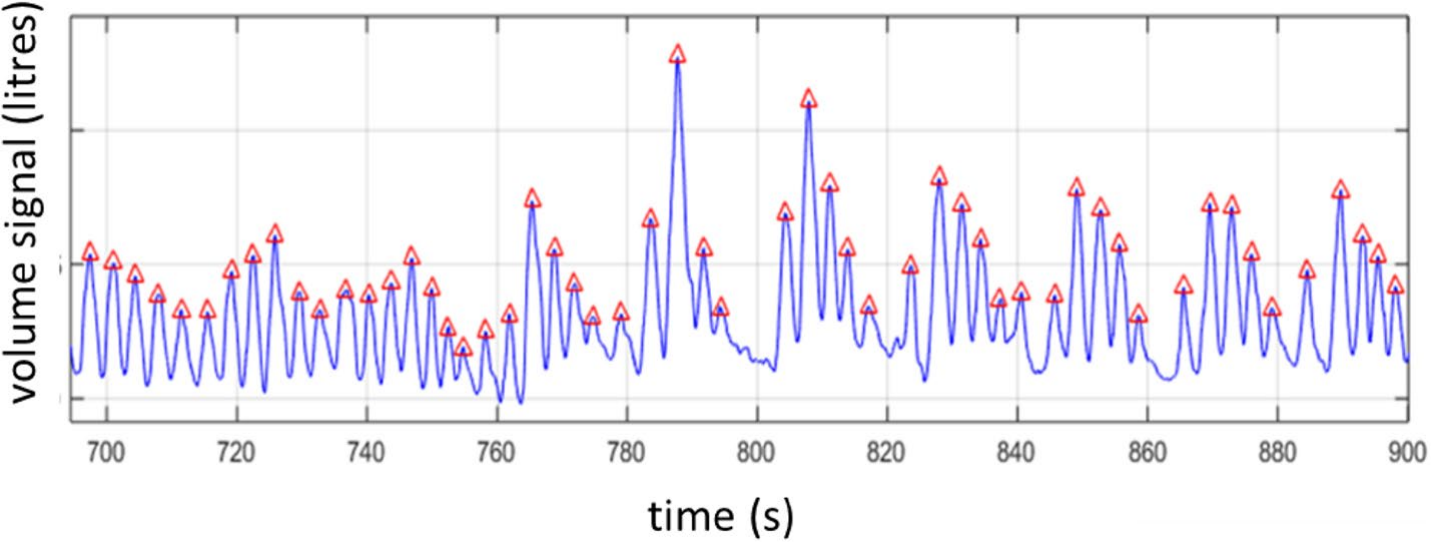
Cyclical respiratory behaviour

Excellent agreement was found between the computed depth sensed respiratory rates and the reference capnograph values, with an overall RMSD of 0.66 bpm. This was achieved with a near zero bias and with 100% uptime. (Even on a per-subject basis, individual RMSDs were all less than 1 bpm.) In addition, the results were achieved over a considerable range or respiratory rates exhibited by the volunteers, (from 6 to 27 breaths per minute). This wide range is indicative of the physiological stress associated with an acute hypoxic challenge and provides a valuable test of the technology. In fact, marked cyclical variations in the respiratory volume signal were observed for some subjects. Figure 7 provides an example this phenomenon. This breathing pattern is a result of hypoxia and the pattern can change as the hypoxia increases. One of the challenges with hypoxia studies is hyperventilation in response to hypoxic gas mixture as the body attempts to maintain as high a PO_2_ as possible in the lungs by reducing CO_2_. The chemoreceptors in the brain detects the decrease in PaCO_2_ and signals to slow down breathing and/or reduce tidal volume, however, the hypoxic drive overrides this signal and make the subject breath deeper. This results in this pattern which is similar in form to Cheyne–Stokes breathing.

A relatively small data set (N = 14) was used in developing the algorithm with no data withheld for separate blind testing. Over-training is a therefore possibility, and the results must be viewed in this context. However, the respiratory signal was manifestly obvious across all patients and a relatively simple algorithm was sufficient to identify individual breaths and produce a respiratory rate. In addition, off-the-shelf depth sensing technology was employed and the equipment is simple to set up with minimal instruction provided to the clinical research staff for its use. (They were asked to set up the camera at chest height within 1 to 2 m of the subject, ensure that the subject was in the frame and turn the acquisition on; with no other intervention or calibration required.)

Motion was relatively restricted as the subjects were seated, attached to a face mask and also had pulse oximeter probes attached to each hand. The participants therefore remained relatively immobile during the acquisition. In clinical practice, however, an algorithm would have to cope with more significant patient motion, including change of posture or position in bed, hand and limb movements, (including hand movements within the line of sight) and interactions with clinical staff. Recently, our group proposed a novel motion protocol for RGB video monitoring including yaw, pitch and roll maneuvers of the head [24]. Although this is at a very early stage and specific to idealized maneuvers for the subject’s head it could perhaps be extended to whole body maneuvers for testing depth-based respiratory technology. However, it is in general very difficult to synthesize the wide variety of complex activities observed in clinical practice and exposing the algorithm to large amounts of patient data acquired from across the spectrum of patient care is ultimately the best way to develop a robust technology.

It is an open question whether depth sensing monitoring could replace other modalities for monitoring respiratory rate in the clinical environment. The technology may be adopted more rapidly in its current state for specific use cases including post-anesthesia respiratory depression indications, neonatal monitoring (where there is a need to avoid excessive contact with the neonatal skin), sleep monitoring and home monitoring of, for example, elderly, post-surgical and/or respiratory patients. The technology also has the advantage of having additional physiological and patient contextual information available from the same modality. These include tidal volume trending, apnea detection, patient activity monitoring, fall detection and bed posture monitoring. A final positive attribute of the technology which should be commented on is its ease of use. Ultimately, the technology should require nothing more than to be aimed at the subject and turned on and it works through patient clothing and bed sheets and with the lights turned off (both of which RGB methods cannot do).

## Conclusion

The results strongly indicate the potential for a robust respiratory rate monitoring technology based on depth sensing camera equipment. Future work should attempt to fully test this technology in a more rigorous fashion through a range of confounders typically exhibited in the clinical setting including motion and interference.

## References

[1] Dahan A, Aarts L, Smith TW (2010). Incidence, reversal, and prevention of opioid-induced respiratory depression. Anesthesiology.

[2] Bergese SD, Mestek ML, Kelley SD, McIntyre R, Uribe AA, Sethi R (2017). Multicenter study validating accuracy of a continuous respiratory rate measurement derived from pulse oximetry: a comparison with capnography. Anesth Analg.

[3] Michard F, Gan TJ, Bellomo R (2019). Protecting ward patients—the case for continuous monitoring. ICU—Manage Pract.

[4] McDuff DJ, Estepp JR, Piasecki AM, Blackford EB. A survey of remote optical photoplethysmographic imaging methods. In: 37th annual international conference of the IEEE engineering in medicine and biology society (EMBC); 2015. pp. 6398–404. 10.1109/EMBC.2015.7319857 26737757

[5] Addison PS, Jacquel D, Foo DM, Antunes A, Borg UR (2017). Video-based physiologic monitoring during an acute hypoxic challenge: heart rate, respiratory rate, and oxygen saturation. Anesth Analgesia.

[6] Li MH, Azadeh Y, Babak T. A non-contact vision-based system for respiratory rate estimation. In: 36th annual international conference of the IEEE engineering in medicine and biology society; 2014. pp. 2119–22. 10.1109/EMBC.2014.6944035 25570403

[7] Scalise L, Ercoli I, Marchionni P, Tomasini EP. Measurement of respiration rate in preterm infants by laser Doppler vibrometry. In: International symposium on medical measurements and applications; 2011. pp. 657–61.

[8] Bu N, Ueno N, Fukuda O. Monitoring of respiration and heartbeat during sleep using a flexible piezoelectric film sensor and empirical mode decomposition. In: 29th annual international conference of the IEEE engineering in medicine and biology society; 2017. pp. 1362–66. 10.1109/IEMBS.2007.4352551 18002217

[9] Droitcour AD, Seto TB, Park BK, Yamada S, Vergara A, El Hourani C, et al. Non-contact respiratory rate measurement validation for hospitalized patients. In: Annual international conference of the IEEE engineering in medicine and biology society; 2009. pp. 4812–15. 10.1109/IEMBS.2009.5332635 PMC4313750 19963625

[10] Al-Khalidi F, Saatchi R, Elphick H, Burke D (2011). An evaluation of thermal imaging based respiration rate monitoring in children. Am J Eng Appl Sci.

[11] Nam Y, Reyes BA, Chon KH (2016). Estimation of respiratory rates using the built-in microphone of a smartphone or headset. IEEE J Biomed Health Inform.

[12] Yu MC, Liou JL, Kuo SW, Lee MS, Hung YP. Noncontact respiratory measurement of volume change using depth camera. In: Annual international conference of the IEEE engineering in medicine and biology society; 2012. pp. 2371–74. 10.1109/EMBC.2012.6346440 23366401

[13] Aoki H, Miyazaki M, Nakamura H, Furukawa R, Sagawa R, Kawasaki H. Non-contact respiration measurement using structured light 3-d sensor. In: 2012 Proceedings of SICE annual conference (SICE); 2012. pp. 614–8.

[14] Seppänen TM, Kananen J, Noponen K, Alho OP, Seppänen T. Accurate measurement of respiratory airflow waveforms using depth data. In: 37th annual international conference of the IEEE engineering in medicine and biology society (EMBC); 2015. pp. 7857–60. 10.1109/EMBC.2015.7320213 26738113

[15] Soleimani V, Mirmehdi M, Damen D, Hannuna S, Camplani M, Viner J, Dodd J. Remote pulmonary function testing using a depth sensor. In: IEEE biomedical circuits and systems conference (BioCAS); 2015. pp. 1–4.

[16] Harte JM, Golby CK, Acosta J, Nash EF, Kiraci E, Williams MA (2016). Chest wall motion analysis in healthy volunteers and adults with cystic fibrosis using a novel Kinect-based motion tracking system. Med Biol Eng Comput.

[17] Transue S, Nguyen P, Vu T, Choi MH. Real-time tidal volume estimation using iso-surface reconstruction. In; IEEE first international conference on connected health: applications, systems and engineering technologies (CHASE); 2016. pp. 209–18.

[18] Bernacchia N, Scalise L, Casacanditella L, Ercoli I, Marchionni P, Tomasini EP. Non contact measurement of heart and respiration rates based on Kinect™. In: IEEE international symposium on medical measurements and applications (MeMeA); 2014. pp. 1–5.

[19] Al-Naji A, Gibson K, Lee SH, Chahl J (2017). Real time apnoea monitoring of children using the Microsoft Kinect sensor: a pilot study. Sensors.

[20] Rezaei B, Lowe J, Yee JR, Porges S, Ostadabbas S. Non-contact automatic respiration monitoring in restrained rodents. In: 2016 38th annual international conference of the IEEE engineering in medicine and biology society (EMBC); 2016. pp. 4946–50. 10.1109/EMBC.2016.7591837 28269378

[21] Martinez M, Stiefelhagen R. Breathing rate monitoring during sleep from a depth camera under real-life conditions. In: IEEE winter conference on applications of computer vision (WACV); 2017. pp. 1168–76.

[22] Cenci A, Liciotti D, Frontoni E, Mancini A, Zingaretti P. Non-contact monitoring of preterm infants using rgb-d camera. In: ASME international design engineering technical conferences and computers and information in engineering conference. American Society of Mechanical Engineers; 2015; pp. V009T07A003-V009T07A003.

[23] Bland J, Altman D (2007). Agreement between methods of measurement with multiple observations per individual. J Pharm Stat.

[24] Addison PS, Foo DMH, Jacquel D. Running wavelet archetype aids the determination of heart rate from the video photoplethysmogram during motion. In: 39th annual international conference of the IEEE engineering in medicine and biology society; 2017. pp. 734–7. 10.1109/EMBC.2017.8036929 29059977

